# Belowground carabid beetle diversity in the western Palaearctic – effects of history and climate on range-restricted taxa (Coleoptera, Carabidae)

**DOI:** 10.3897/zookeys.100.1540

**Published:** 2011-05-20

**Authors:** Andreas Schuldt, Thorsten Assmann

**Affiliations:** Leuphana University Lüneburg, Institute of Ecology and Environmental Chemistry, Scharnhorststr. 1, D-21335 Lüneburg, Germany

**Keywords:** Cave fauna, endogeic, glaciations, geographic range, insects, latitudinal gradient, macroecology, permafrost, subterranean

## Abstract

Broad-scale patterns of subterranean diversity are a fascinating but neglected part of biodiversity research. Carabid beetles adapted to belowground habitats form a particularly species-rich part of the subterranean fauna. We studied large-scale diversity patterns of these belowground carabids across the western Palaearctic and evaluated potential impacts of historical and contemporary environmental conditions on the distribution of these taxa, using available species richness and environmental data at country level. Regression modelling and variation partitioning showed a strong relationship between species richness and range in elevation. Potential effects of climatic variables, mainly those related to ambient energy input, were much weaker. We discuss the implications of this combination of effects, which suggests, concordant with the absence of subterranean carabids in northern and highest richness in southern Europe, a strong prevailing influence of historical processes on current richness distributions of these taxa. Previous studies did not provide clear indications for such an influence. In contrast to more mobile and widespread carabid beetles, dispersal limitation due to high adaptation of belowground carabids to subterranean habitats has probably hindered their re-colonization of former permafrost and glaciated regions. Hotspots of highest belowground diversity are located in regions with an assumed long-term stability of environmental conditions, correlating with patterns of other dispersal-limited taxa such as many endemic plants. Our study provides important new information in the discussion of potential determinants of the distinct geographic patterns of belowground diversity. Moreover, it contributes to a better understanding of range size related differences previously found in the distribution of diversity and environmental dependencies of widespread and range-restricted species within the highly diverse carabid beetles.

## Introduction

Studies on the spatial patterning of biodiversity and its potential determinants have gained much attention over the last decades, especially in light of global change and its assumed effects on the distribution and survival of many species (Kerr et al. 2007). In this regard, the diversity of belowground habitats has been studied only poorly, even though it comprises many rarely recorded and highly adapted species (Culver et al. 2006). These habitats are characterized by much lower temporal variability of the physical environment than aboveground habitats (Wilkens et al. 2000; Assmann et al. 2010). Still, dependencies on environmental factors that have been identified as potential drivers of the distribution of diversity of many aboveground organism groups are being assumed also for subterranean diversity (Culver et al. 2006; Zagmajster et al. 2008). Especially productivity of the aboveground habitats, which again is determined by an interplay of energy input and water availability (Hawkins et al. 2003), has repeatedly been suggested as a possible factor influencing diversity in these subterranean habitats, which are often considered (and this particularly applies to caves) to be to a large part dependent on allochtonous input of resources (Gers 1998; Culver et al. 2006). Such a dependency on environmental factors could have far-reaching consequences for the strongly dispersal-limited Belowground taxa regarding future shifts in environmental conditions due to climate change (Sharratt et al. 2000).

However, reduced dispersal abilities due to their strong habitat specialization also suggest that especially processes and environmental conditions over historical times have played important roles in the survival and present distribution of these species. Regions with high numbers of subterranean species have probably been subject to lower variability in environmental conditions over long time periods and a higher persistence of ecosystems than other western Palaearctic regions (Casazza et al. 2008; Assmann et al. 2010).

About 50% of the terrestrial fauna in subterranean habitats are beetles (Zagmajster et al. 2008). With more than 1,000 troglobiont and endogeic species described so far, carabid beetles make up a large part of this subterranean fauna in the western Palaearctic, which harbours one of the hotspots of highest diversity of terrestrial troglobites in the northern hemisphere (Casale et al. 1998; Culver and Sket 2000; Culver et al. 2006; Assmann et al. 2010). Carabids are thus also well suited to provide important insights into aspects of faunal diversity of subterranean habitats. Moreover, results from the study of the highly diverse subterranean carabid fauna also have implications for the understanding of general patterns in the distribution of biodiversity over large geographic extents. Schuldt and Assmann (2009) found differences in the potential impact of current climate and historical processes on overall diversity and that of widespread and endemic species of carabid beetles in the western Palaearctic, probably due to differences in the dispersal abilities of these taxa. Comparison of these patterns to those of the strongly range-restricted carabids living in belowground habitats can help to extend our understanding of the distribution of diversity and the possible mechanisms behind such range-size dependent diversity patterns.

The aim of our study was to analyse spatial patterns in the species richness of belowground carabids and their potential environmental determinants on a large scale across Europe and North Africa. Distinguishing between the carabid fauna of deeper soil horizons, beetles of the so-called superficial underground compartment (“milieu souterrain superficiel”, MSS; Juberthie 1979) as a macroporal system in rocky material, and obligate cave-dwellers is not possible for all regions of the western Palaearctic, as species known as specialized cave dwellers have also been recorded in the MSS (e.g., *Aphaenopidius kamnikensis* Drovenik 1987, a carabid until recently known to occur only in caves; Drovenik et al. 2007). Thus, in our analysis we combine all species from these systems with an obligate subterranean way of living and use the term “belowground fauna” for these taxa from hereon. Species with the ability to fly and also occurring in aboveground habitats (e.g., species of *Limnastis*) were not treated as part of the belowground fauna.

Analysing country-level species and environmental data, we hypothesize weak links between belowground diversity and current climatic conditions and a strong signal of history contained in broad-scale distribution patterns of belowground carabids. This would support and help to explain the previous findings concerning range size dependent differences between total, widespread and endemic richness of carabids in the response to large-scale environmental conditions (Schuldt and Assmann 2009).

## Methods

### Species and environmental data

Species numbers of carabid beetles were extracted from Löbl and Smetana (2003) for 39 countries of the western Palaearctic. The distribution of diversity for most invertebrates is not well documented at smaller scales over such large geographic extents, which hinders analysis on a more detailed scale or assigning reliable data to equal-area grids (Baselga 2008; Hortal 2008). In contrast, country-level data for carabid beetles in the western Palaearctic is quite comprehensive and allows accurate analysis of macroecological patterns for such a highly diverse insect taxon (Schuldt and Assmann 2009; Schuldt et al. 2009). In our analyses, we excluded Iceland due to its strong insularity as well as Andorra, Liechtenstein and Luxembourg because of their small country size. All species with a strict subterranean or cave-dwelling lifecycle, as documented in the literature and indicated by reduced and missing eyes, were classified as belowground species (see Table S1 in Supplementary Material for a list of the genera included). While total species richness is well documented for most western Palaearctic countries (Schuldt and Assmann 2009), new species are still being recorded from subterranean habitats in the most species-rich southern European countries. However, this does not affect the overall spatial pattern of species richness, neither for total nor for belowground carabid richness. We used patterns of total species richness of all carabids and the richness of widespread and endemic carabids (all extracted from Löbl and Smetana 2003) for comparison with distribution patterns of belowground beetles. Widespread (range sizes >6 x 105 km²) and endemic carabids (range sizes <6 x 105 km²) were classified following the definition of Lumaret and Lobo (1996). As we were also interested in the relationship with species richness of vascular plants, we compiled data for this taxon from Walter and Gillett (1998), Groombridge and Jenkins (2002) and CBD National Reports (www.cbd.in/countries).

For our analyses we used a set of environmental variables related to recently intensively discussed hypotheses (Willig et al. 2003) on the influence of climatic and topographic factors on the spatial distribution of species richness (see Table 1 for a complete list of variables used). Climate data were obtained as country-level averages of high-resolution data from Mitchell (2002) and comprised mean annual temperature, mean temperature of the coldest and the warmest month, temperature seasonality (difference between warmest and coldest month), mean annual precipitation, mean precipitation from March to November, seasonality in precipitation (difference between wettest and driest month) and the number of days with frost. Additionally, mean annual potential and actual evapotranspiration (PET and AET) were calculated using Thornthwaite’s method (Thornthwaite and Mather 1963; 1964; Black 2007). AET is often considered a surrogate measure for productivity (Hawkins et al. 2003). Finally, range in elevation (i.e. the difference between the highest and lowest elevation within each sampling unit) was compiled from CIA (2008) as a measure of both habitat heterogeneity as well as prevailing signals of evolutionary and historical processes (Schuldt and Assmann 2009). From the same source, we extracted midpoint latitude and longitude of the countries to quantify the spatial dimension of richness distributions.

### Statistical analyses

Environmental correlates of species richness of belowground carabids were first analysed in regressions with single environmental variables. Second- or third-order polynomials were added to the centred predictor variables in case of significant non-linear relationships. Species richness and country area were log10-transformed to normalize distributions.

We then used regression modelling to assess the separate and combined impact of three different sets of predictor variables (spatial, topographic and climatic) on the richness pattern of belowground carabids. Spatial, topographic and climatic factors might explain similar proportions of the variability in the observed richness patterns. Our approach allows us to handle the non-independence of predictor variables, which might show the same autocorrelated pattern but relate to different conceptual frameworks in the explanation of diversity patterns, and thus to identify the isolated influence of different sets of explanatory variables (Baselga 2008; Hortal et al. 2008). For each set, we computed stepwise regression models with backward elimination, excluding variables that caused low tolerance (<0.1) due to high multicollinearity (r >0.9) with other variables in the predictor sets (Quinn and Keough 2002). Variation partitioning was used to assess the independent (i.e., purely spatial, topographic and climatic) and shared (spatially structured and co-varying) effects of the three predictor sets on Belowground carabid richness in a combined model (Legendre and Legendre 1998; Hortal et al. 2008). We included area as a co-variable into the analyses to account for differences in country size.

Spatial autocorrelation can inflate statistical errors in analyses of geographic diversity patterns (Diniz-Filho et al. 2003). To account for this, we recalculated significance of regressions using spatially corrected degrees of freedom by correlating observed and predicted values of regressions (Qian et al. 2007) according to the modified t-test by Dutilleul (1993). Additionally, we checked the adequacy of our non-spatial regression on climate and topography to explain the spatial structure in the belowground carabid data by generating a correlogram with Moran’s I coefficients, which show the reduction in spatial autocorrelation after fitting the regression model (Diniz-Filho et al. 2003). Moran’s I values of zero indicate absence of spatial autocorrelation, whereas larger or smaller coefficients (usually ranging between +1 and -1) show the degree of positive or negative autocorrelation between neighbouring sampling units. Non-significant values of Moran’s I coefficients after fitting the explanatory variables indicate that the variables selected well account for the spatial pattern in the richness data (Diniz-Filho et al. 2003).

All statistical analyses were performed with SPSS 15.0 for Windows (SPSS, Chicago) and SAM 2.0 (Rangel et al. 2006).

**Table 1.** Results (coefficients of determination, F-values, degrees of freedom and spatially corrected probabilities) of **a** regressions of belowground carabid species richness against single environmental variables and **b** regression modelling of species richness of belowground carabid beetles in the western Palaearctic.

		*Model (function)*	*R²adj*	*F*	*DF*	*p*
a) single regressions						
	Latitude (decimal degrees)	*lat-lat²+lat³*	0.51	12.6	3, 35	0.012
	Longitude (decimal degrees)	*n.s.*				
	Area (km²) (log10)	*n.s.*				
	Elevation range (m)	*elev (+)*	0.52	38.7	1, 37	<0.001
	Mean annual temperature (°C)	*mean_temp-mean_temp²*	0.26	6.4	2, 36	0.042
	Mean temperature coldest month (°C)	*n.s.*				
	Mean temperature warmest month (°C)	*mean_warm-mean_warm²*	0.24	5.8	2, 36	0.052
	Temperature seasonality (°C)	*n.s.*				
	Mean annual precipitation (mm)	*n.s.*				
	Mean precipitation March-November (mm)	*n.s.*				
	Seasonality precipitation (mm)	*n.s.*				
	Potential evapotranspiration (mm/yr)	*PET-PET²*	0.38	11.1	2, 36	0.020
	Actual evapotranspiration (mm/yr)	*n.s.*				
	Frost frequency (days)	*frost (-)*	0.14	5.0	1, 37	0.048
b) regression modelling						
	Spatial (S)	*lat-lat²+lat³*	0.51	12.6	3, 35	0.012
	Topographic (T)	*elev*	0.52	38.7	1, 37	<0.001
	Climatic (C)	*PET-PET²*	0.38	11.1	2, 36	0.020
	Combined (T+C)	*elev; PET-PET²*	0.69	26.5	3, 35	<0.001
	Total (S+T+C)	*lat-lat²+lat³; elev; PET-PET²*	0.68	13.5	6, 32	<0.001

Appendix 1, Figure S1 and Table S1.pdf – Spatial correlogram and list of Belowground genera.

## Results

**Figure 1.**
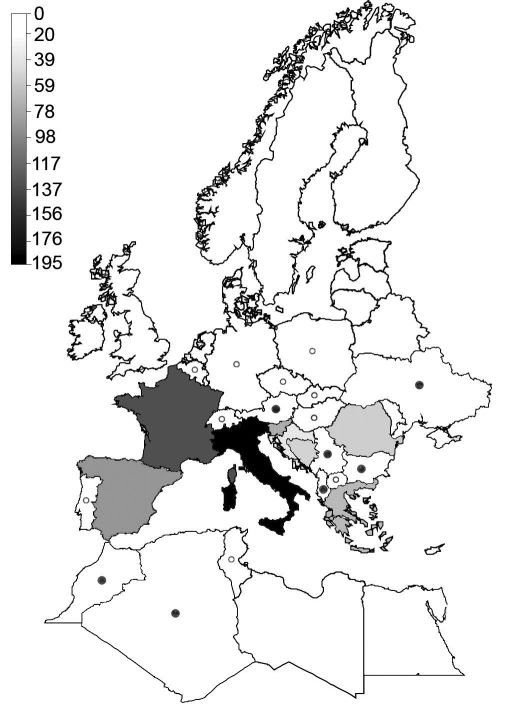
Distribution of species richness of belowground carabid beetles across the western Palaearctic, based on Löbl and Smetana (2003). Shadings and symbols indicate the number of species recorded for each country. Countries with 11–20 subterranean species are marked by a filled circle, countries with 1–10 species by an open circle. Countries for which no subterranean species have been recorded are white and without a symbol.

The distribution of species richness of carabids adapted to belowground habitats showed a clear and significant latitudinal gradient across the western Palaearctic (Table 1). Richness was highest in southern European regions (Fig. 1). This especially applies to Italy, which featured highest species numbers. Larger countries such as France and Spain had lower numbers and even for the Balkan Peninsula, species numbers were lower even when an area of comparable size and latitudinal extent was considered (i.e., Albania, Bosnia-Herzegovina, Greece, Macedonia and Serbia-Montenegro, which as a whole is slightly larger than Italy but harbours only 146 species as compared to 195 species for Italy). Even with Romania, Bulgaria and Croatia added to this latter region, which increases the area to more than twice the size of Italy, this region harbours only 36 more species than Italy.

**Figure 2.**
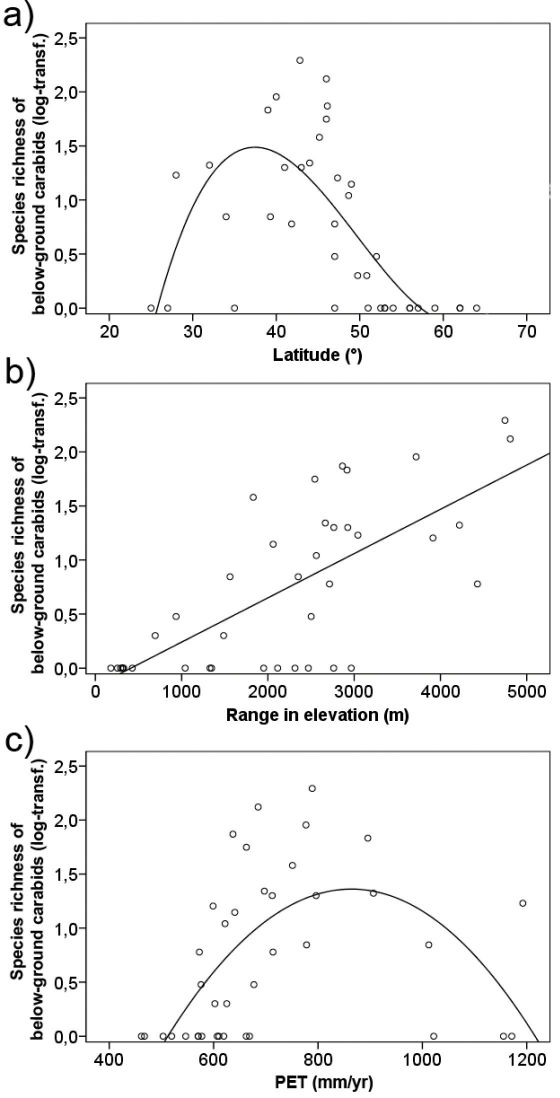
Relationship between species richness of belowground carabid beetles (log10-transformed) and **a** latitude (R²adj.=0.51; p=0.012), **b** range in elevation (i.e., topograohic variability; R²adj.=0.52; p<0.001) and **c** annual potential evapotranspiration (R²adj.=0.38; p=0.020) in the western Palaearctic.

Species numbers decreased towards northern Europe and North Africa (Fig. 2a) and most countries completely lacked belowground carabids, especially in the northern part of Europe (Fig. 1). In contrast to latitude, subterranean carabids did not show a significant relationship with longitude. Species numbers were also not significantly related to the size of the countries analysed (Table 1a). Several environmental variables were correlated with species richness of belowground carabids. Richness showed a linear increase with and was most strongly (R²=0.52; p<0.001) related to range in elevation (Table 1a, Fig. 2b). Variables related to ambient energy input (PET, mean annual and mean temperature of the warmest month) showed a hump-shaped relationship with species richness (R² between 0.24 and 0.38; p≤0.052), which increased up to a certain level with increasing available energy and decreased again at highest levels of energy input (Table 1, Fig. 2c). Frost frequency was negatively related to species richness, whereas precipitation measures and AET were not significantly related to belowground carabid diversity (Table 1).

**Figure 3.**
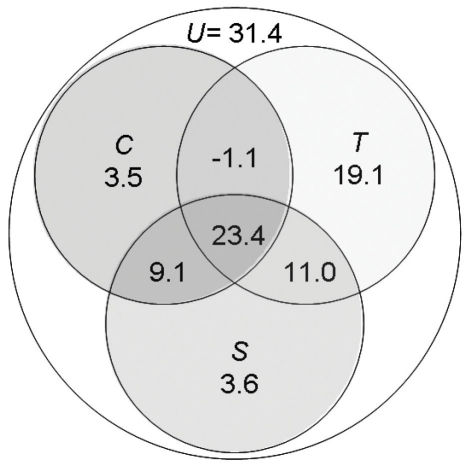
Partitioning of variation from regression modelling for species richness of belowground carabids. Values give the % of the total variation independent and shared effects of spatial (**S**), topographic (**T**) and climate (**C**) models account for in the explanation of richness patterns. **U** is the unexplained variation.

Regression modelling identified a polynomial term of latitude, the linear measure of elevation range and a quadratic term of PET as the best predictors of spatial, topographic and climatic models for species richness of belowground carabids (Table 1b). A combined model of elevation range and PET explained 69% of the variability in the carabid data. It removed all significant spatial autocorrelation from the carabid data. Moran’s I coefficients in a spatial correlogram over ten distance classes were all close to zero and non-significant after fitting the model (see Appendix 1, Figure S1: Spatial correlogram). This shows that these variables quite well account for the spatial structure in the distribution of subterranean carabid diversity and that modelling results are not affected by spatial autocorrelation (Diniz-Filho et al. 2003). The total model, adding a spatial component to these variables, did not increase the goodness of fit and explained 68% of the data variability (Table 1b). Variation partitioning showed that range in elevation had by far the strongest independent effect on species richness of belowground carabids, accounting for 19% of the explained variance. Together with the spatially structured effect of elevation range, this factor explained 30.1% of the carabid data variability (Fig. 3). In comparison, independent spatial and climatic as well as spatially structured climatic effects were weak. The shared variation for all three components together, i.e. spatially structured climatic and topographic effects, was 23.4% (Fig. 3).

Richness of Belowground carabids was strongly correlated with total species richness of carabid beetles (Pearson’ r=0.76; p<0.001, corrected for spatial autocorrelation), less strongly with richness of widespread species (r=0.63; p=0.001) and most strongly with richness of endemic carabids (r=0.87; p<0.001). It was also highly correlated with species richness of vascular plants (r=0.86; p<0.001) across the western Palaearctic.

## Discussion

The spatial analysis of belowground carabids clearly identifies southern Europe, and especially Italy, as the region with highest species richness in the western Palaearctic. Generally, this region has been classified as one of the hotspots of overall terrestrial troglobiont diversity based on the comparison of local species numbers from single caves (Vigna Taglianti 1982; Culver and Sket 2000) and from the small-scale study of selected regions across Europe (Culver et al. 2006). Our study extends this knowledge to the regional scale for one of the most species-rich terrestrial belowground taxa using a spatially and temporally comprehensive dataset. So far, the diversity of separate subterranean groups has scarcely been studied in detail (Zagmajster et al. 2008). Moreover, in contrast to most previous studies focusing on obligate cave-dwellers, our study comprises a broader range of subterranean species, including those living in the superficial underground compartment (MSS; Juberthie 1979), and thus provides a more general picture of belowground diversity patterns.

Species richness of carabid beetles adapted to belowground habitats significantly and most strongly co-varied with range in elevation. To a certain extent, this might reflect effects of habitat heterogeneity and availability (Kerr and Packer 1997; Rahbek and Graves 2001), as mountainous regions are likely to feature extensive cave and karst systems. However, large karst areas also occur in regions which harbour only few or no belowground carabids at all (e.g., Belgium, England, Switzerland; see also Culver et al. 2006). Rather, our findings indicate a strong effect of historical processes on the present distribution patterns of belowground beetles. Regions with high altitudinal ranges can promote diversification processes through isolation and segregation along altitudinal gradients in environmental conditions (Jetz et al. 2004; Rahbek et al. 2007; see also Casale 2009). Moreover, topographically highly variable regions allow species to effectively compensate climatic shifts (Hewitt 1999) and not surprisingly, the highest belowground diversity of carabids was found in mountainous regions where probably the southern refugia of many taxa during the last ice age were located (Hewitt 1999; Casazza et al. 2008; Drees et al. 2010). The latitudinal pattern with a steep decrease in richness towards northern Europe, combined with relatively low effects of climate variables, yields further information regarding historical impacts. Due to their very specific habitat requirements and morphological adaptations, carabid beetles of belowground environments are strongly limited in their dispersal (Lamoreux 2004; Assmann et al. 2010). The lack of belowground species in central and northern Europe, despite suitable habitats, might be attributed to extinctions during Pleistocene glaciations and the inability of range-restricted taxa to re-colonize these regions (see also Schuldt and Assmann 2009). For many range-restricted and even for widespread vascular plants, effects of dispersal limitation on the current distribution across Europe have been suggested (Svenning and Skov 2007; Svenning et al. 2008). A strong correlation between richness patterns of belowground carabids and vascular plants might indicate a similar historical signal prevailing in the distribution patterns of both taxa (cf. Hewitt 1999).

Concerning the distribution of hotspots of overall troglobiont diversity in Europe, Culver et al. (2006) found weak support for an influence of Pleistocene glaciations. Similarly, belowground carabids are also missing south of the former boundary of the Pleistocene ice sheet. However, the coarse-scale distribution of belowground carabid beetle diversity seems to largely conform to patterns postulated by Holdhaus (1954), who hypothesized that the occurrence of terrestrial cave fauna in Europe has been influenced by the spatial extent of permafrost soil, stretching much farther south than the ice sheet. According to his studies, the northern limit of these taxa runs along a line (the “Holdhaus-line”) from the southern part of the Alps eastwards to the Carpathians (Holdhaus 1954; Drees et al. 2010). Concordance between the distribution of several groups of blind carabids and the theory of Holdhaus has also been found by Drees et al. (2010). Further re-examination on a more detailed scale will be needed to accurately evaluate these findings in light of the numerous new records of subterranean taxa from the last decades. While some species are considered to have survived in isolated refuges north of the Holdhaus-line (Holdhaus 1954), further deviations from the general pattern might be explained by postglacial range expansions (Drees et al. 2010). In contrast, an *Anillus* species recorded in park locations of Belgium (Desender 1986) and Germany (Malzacher 2000) was probably introduced with soil from the root system of trees imported from southern Europe. The survival of this species shows that suitable habitats also exist now in northern regions of Europe and can be seen as further evidence for strong effects of dispersal limitation on re-colonization processes in the western Palaearctic (Drees et al. 2010).

As mentioned above, the influence of dispersal limitation might also become evident from the fact that current climate accounted for much smaller amounts of explained variation than elevation range in both single regressions as well as in regression modelling. Species with well-developed dispersal abilities are assumed to have tracked post-Pleistocene climate changes to a certain degree, and high co-variation between species richness of many taxa and climatic variables support this view (reviewed by Hawkins et al. 2003). Within the highly diverse carabid beetles, the same is true for the richness of more mobile, widespread species, which is strongly related to climatic variables and much less to elevation range (Schuldt and Assmann 2009). In contrast, more range-restricted endemic species show the opposite pattern, with a strong impact of topographic variability and low influence of current climate pointing to prevailing effects of historical processes on distribution patterns (Schuldt and Assmann 2009). Our results for belowground beetles as part of the range-restricted carabids strongly support these findings and thus provide further insight into potential mechanisms underlying spatial distributions of diversity. They show that the significance of historical processes in explaining contemporary richness distributions might increase as dispersal abilities of the species decrease. In this respect, belowground carabids have a strong impact on overall patterns of range-restricted (endemic) species. A larger influence of climate on these overall patterns of endemics (Schuldt and Assmann 2009) compared to belowground diversity, even though still secondary to effects of elevation range, indicates that postglacial range expansions are easier in above- than in the often spatially isolated belowground habitats (Porter 2007).

At least at the coarse scale of our analysis, we did not find evidence for the assumptions of Culver et al. (2006) that centres of highest belowground diversity might be located in regions of long-term high aboveground productivity. Current and past climate are correlated (Araújo et al. 2008) and AET as a measure of productivity (Hawkins et al. 2003) was not related to richness patterns of belowground carabids in our study. Rather, an influence of variables representing ambient energy input (temperature, PET) and the potential effects related to topographical variability suggest that in the western Palaearctic highest richness of these taxa is determined by historical/evolutionary processes and a general long-term stability of environmental conditions (temperature) which supported survival and, especially regarding the strong signal of history, promoted diversification processes in belowground habitats (e.g., through isolation from other similar habitat patches or other phenomena relevant for evolutionary processes; Casale and Vigna Taglianti 2005).

## Appendix 1

Spatial correlogram and list of Belowground genera. (doi: 10.3897/zookeys.100.1540.app) File format: Adobe PDF (.pdf).

Copyright notice: This dataset is made available under the Open Database License (http://opendatacommons.org/licenses/odbl/1.0/). The Open Database License (ODbL) is a license agreement intended to allow users to freely share, modify, and use this Dataset while maintaining this same freedom for others, provided that the original source and author(s) are credited.
